# PROTOCOL: Food Environment, Food Choice, Diets, and Nutrition Outcomes of Pastoralists in Africa: Scoping Review Protocol

**DOI:** 10.1002/cl2.70030

**Published:** 2025-03-10

**Authors:** Esther Omosa, Francoise Cattaneo, Matthew Kibbee, Paula Dominguez‐Salas, Natasha Bishop, Inge D. Brouwer

**Affiliations:** ^1^ Wageningen University and Research Wageningen Netherlands; ^2^ International Livestock Research Institute Nairobi Kenya; ^3^ Cornell University Ithaca USA; ^4^ Natural Resource Institute University of Greenwich Kent UK; ^5^ International Food Policy Research Institute Washington DC USA

**Keywords:** diet, food choice, food environment, nutrition outcome, pastoralists

## Abstract

This is the protocol for a Campbell scoping review. The objectives are as follows: (i) To define and characterize the food environment of pastoralists in Africa; (ii) To identify the domains of the food environment that have been studied in pastoralist settings in Africa; (iii) To assess the relationship between the food environment and food choice, dietary intake, and nutrition outcomes among pastoralists in Africa; and (iv) To map the study designs, methods, and geographical coverage of the studies.

## 1 Introduction

The food environment is an important interface within the wider food system, where individuals interact with food sources to acquire and consume foods. It drives the consumers' decisions on what to acquire, prepare, and consume and it has implications on diet and nutrition outcomes (Downs et al. 2020; Downs et al. 2022; HLPE 2017; Rankin et al. 2018; Toure et al. 2021; Turner et al. 2018). As a linkage between the food system and individual diets, the market‐based influences of the food environment interact with individuals to enable or constrain people's food acquisition, consumption, and related nutrition and health outcomes (Toure et al. 2021, 2020). The food environment incorporates personal (food accessibility, affordability, convenience, and desirability) and external (availability, prices, vendor and product properties, and marketing and promotion) domains (Turner et al. 2020). It also incorporates built (formal and informal) and natural (domesticated and wild) spaces, and the infrastructure surrounding the consumer (Downs et al. 2020).

Malnutrition disproportionately affects pastoralists who live mainly in the marginal rangelands and rely on their livestock for livelihood and food. In Africa for instance, cases of over 10% points more acute malnutrition levels are observed among pastoralists compared to other communities (Chotard et al. 2010). Micronutrient deficiencies are highest in Africa compared to other regions globally (Stevens et al. 2022). Women and children in pastoralist communities are most affected mainly due to consumption of less diverse foods and in insufficient quantities (Gebre et al. 2019). Deficiencies for important micronutrients, such as iron, folate, zinc, iodine, and vitamins B12 and D, still remain high (UNICEF 2021); for example, about one in three women (517 million women) worldwide are anemic. The WHO (2024) report indicates that anemia in women has increased worldwide, after 20 years of stagnation. Pastoralist women are assumed to be at a greater risk, but information is missing (Mshanga et al. 2020). Malnourished women not only have a higher risk of sickness and death but suboptimal pregnancy outcomes affect their children's cognitive development, quality and length of life, especially during the first 2 years of life, limiting attainment of full potential in adult life (Black et al. 2013; UNICEF 2021).

The diet of pastoralists is often poor and characterized by low diversity and inadequate quantities (Abu‐Saad and Fraser 2010). Risks related to environmental, demographic, and economic factors are important drivers of the food system and have implications on food choice, diet consumption, and nutrition outcomes (Kimaro et al. 2018; Uddin and Kebreab 2020). It is already projected that the current food systems cannot meet the dietary needs of an increasing pastoralist population relying on constrained natural resources, especially land and water that are under threat by climate change (Uddin and Kebreab 2020). These factors affect food environment domains, such as types of food available, affordability, accessibility, and quality, which in turn influence choice of foods consumed and nutrition outcomes. Evidence from pastoralist communities in Africa indicates that food insecurity is high, where food availability, access, affordability, and stability is often unassured most of the time (COMESA 2021).

Food choice is the process through which consumers select, acquire, prepare, consume, and dispose of food, arising from an interaction of enabling or constraining factors in the food environment (Shepherd and Raats 2006). Food behavior is influenced by determinants that act at various levels. The socioecological model argues that these determinants can be individual, institutional or organizational, community, and public policy (Stokols 1996), but important dimensions related to physical environment and sociocultural contexts do influence food choice (McLaren and Hawe 2005). At an individual level, factors such as consumer preference, socioeconomic conditions, social environment, psychological factors, and cultural relevance (Blake et al. 2021) interrelate with the food environment to determine what, where, when, how, and why certain food choices are made (Downs et al. 2020).

There is a need for more evidence on the food environment in low‐ and middle‐Income countries (LMICs) to inform interventions, for instance in pastoralist settings where malnutrition is a big concern. It is no doubt that the current nutrition transition experienced in LMICs due to changes in economic development, globalization, technology advancement, and climate change, is shaping the food environment in these countries (Constantinides et al. 2021). There is a substantial body of evidence from high‐income countries (HICs), and whilst the evidence base from food environment research in LMICs is rapidly emerging, there has been little attention in low‐income countries and rural areas to date (Constantinides et al. 2021; Turner et al. 2020). A systematic review conducted by Turner et al. (2020) revealed that few studies in LMICs have assessed associations between the food environment and diets, nutrition, and health outcomes. More studies have assessed associations between food availability and diet outcomes, very few have assessed associations between food availability and nutrition and health outcomes. However, some concerns arise as to whether this association is worthwhile to study because of multiple confounding factors. For instance, some studies do not support use of stunting as an appropriate outcome of agricultural or food system investments due to the complexity in causality and attribution (Leroy et al. 2022). There are suggestions that reducing food prices and increasing affordability, availability, and quality and safety of foods are potential starting points in supporting consumption of healthy diets (Constantinides et al. 2021). A healthy diet is one which promotes growth and development and prevents all forms of malnutrition. Malnutrition refers to undernutrition—wasting, stunting, underweight, deficiencies in vitamins or minerals—and obesity as well as dietary factors that increase the risk of non‐communicable diseases (NCDs), such as heart disease, stroke, diabetes, and certain cancers.

Further, little is understood about the relationship between food environment and individual food choices. Constantinides et al. (2021) analyzed the relationship between food environment and food choices in various contexts and concluded that understanding the food environments and individual‐level drivers of food choice in various contexts is an important step in designing policies, initiatives, and interventions for healthy diets.

The uniqueness of pastoralist food environment contexts has not been considered in all these studies, yet pastoralists continue to experience high levels of malnutrition alongside some investments to address food production, diets, and malnutrition with minimum or no impact (Bekele et al. 2021).

To comprehensively address diet quality as a major cause of malnutrition in all its forms in pastoralist settings, a clear characterization of the food environment domains and how the food environment influences food choice, consumption, and nutrition outcomes is necessary. Further, it is important to understand the designs, methods and the geographical coverage of the studies. Such information is best addressed using a scoping review as it meets the following four indications guided by Munn et al. (2018): identification of the types of existing evidence in a certain discipline, clarification of key concepts, identification of characteristics associated with a concept, and identification and analysis of knowledge gaps.

The primary research question for this scoping review is, “What are the key characteristics of the food environment among pastoralists in Africa, and how do they influence food choice, dietary intake, and nutrition outcomes among this population?”

This scoping review therefore aims to provide information on the food environment domains, study approaches and their geographical coverage, food choice, dietary intake, and nutrition outcomes of pastoralists in rural Africa. This information would be useful for designing a pastoralist food environment framework to identify food environment options to improve consumption of healthy diets for better nutritional outcomes. Further, the findings will identify and analyze research gaps on the food environment in pastoralist settings in Africa.

## 2 Methods and Analysis

### 2.1 Protocol Structure

Items in this protocol are reported according to the Preferred Reporting Items for Systematic reviews and Meta‐Analyses Extension for Scoping Reviews (PRISMA‐ScR) guidelines. Our methods will follow the conduct guidelines of the updated Methodological Expectations of Campbell Collaboration Intervention Reviews (MECCIR; Aloe et al. 2024; Young et al. 2024) and the framework process by (Arksey and O'Malley 2005), which comprises five stages, namely (i) identifying the research question; (ii) identifying relevant studies; (iii) study selection; (iv) charting the data; and (v) collating, summarizing, and reporting the results.

The scoping review will utilize the population, concepts, and context (PCC) approach to direct research questions, inclusion, and exclusion criteria (Peters et al. 2020, 2021), as summarized in the Table 1 below.

**Table 1:** Population, concepts, and context approach for the scoping review.

Concepts	Subcategory	Include	Exclude
Population	Pastoralists diet.	Male and female, aged 2 years and above, living in pastoralist settings.	Living in the pastoralist region, not keeping animals, at all in any time of the year.
Concepts	Food environment domains, including availability, accessibility, price, vendor and product properties, desirability marketing, and information. Food choice motives, dietary intake, dietary practices, dietary habits, and nutrition outcomes Food environment interventions.	One or more food environment domains that influences food choice, dietary intake, or nutrition outcome. Dietary assessment methods e.g., 24‐h‐recalls and food frequency questionnaires. Studies reporting on food environment and associations with food choice, dietary intake, and nutrition outcomes, including for children above 2 years of age and adult women.	Studies not including any food environment domain. Studies that do not report any relevant outcome, such as food choice, dietary intake, or nutrition outcome.
Context	All countries or regions in Africa	Any country in Africa with a specific focus on Kenya. Eastern Africa, Western Africa, Southern Africa, North African countries, and Central Africa.	Countries outside Africa.
Type of studies	Design	Quantitative and qualitative studies: Observational studies Cross‐sectional studies, Intervention studies, Ethnographic research. Reviews: Systematic, Scoping, Umbrella, and Narrative conference proceedings. Concept analyses. Unpublished/non‐peer reviewed literature. Theses and dissertations. Grey literature will be sourced from FAOLEX (Food and Agriculture Organization of the United Nations: https://www.fao.org/faolex/en/, Gardian (CGIAR: https://gardian.bigdata.cgiar.org/#/, and the International Development Research Centre (IDRC: https://idrc.ca/en/search).	Blogs Opinion pieces Editorials.
Publication year	Year	January 1, 2000, to present (2024).	Any period earlier than January 1, 2000, and after the date of the last search.
Language of publication	Language	English	Any other language that is not English.

Given that the food environment research, including food environment domains, is a recently evolving concept hinged on the food system principles, we proposed to have the year of publication to start from January 1, 2000 as we do not expect to get relevant articles earlier than 2000.

Step 1: Identifying research questions

The specific objectives for this scoping review are: (i) to define and characterize the food environment of pastoralists in Africa, (ii) identify the domains of the food environment that have been studied in pastoralist settings, (iii) to assess the relationship between the food environment and food choice, dietary intake, and nutrition outcomes among pastoralists in Africa, and (iv) to map the study designs, methods, and geographical coverage of the studies.

The following review questions will guide this scoping review.

1. How is the food environment defined and characterized in pastoralist settings in Africa?
2. What domains of the food environment have been studied in pastoralist settings in Africa?
3. What is the relationship between the food environment and food choice, dietary intake, and nutrition outcomes among pastoralists in Africa?
4. What study designs and methods are used and their geographical distribution?

Step 2: Identifying relevant studies

A prior search on food environment, food choice, dietary intake, and nutrition outcomes in pastoralist settings was conducted in Cochrane Database of systematic reviews, JBI evidence synthesis, Web of Science, Scopus, ScienceDirect, and PROSPERO but did not yield planned reviews on the pastoralist food environment or association of food environment with food choice, dietary intake, and nutrition outcomes in pastoralist settings. One identified review aimed to provide evidence on the characteristics of the food environment that influence women's food acquisition practices and dietary intake in LMICs (O'Meara et al. 2023). However, does not cover nutrition outcomes of all pastoralists households. This therefore makes our study different as it focuses on pastoralist household members above 2 years of age, including nutrition outcomes, and in Africa only.

### 2.2 Search Strategy

The food environment framework adapted from (Downs et al. 2020; HLPE 2017; Turner et al. 2018) will guide the categorization of different domains of the food environment.

In the search strategy, three steps as outlined by Peters et al. (2020, 2021) were followed to develop a full search strategy. Research librarians specializing in evidence synthesis developed a search strategy using text words found in the titles and abstracts of a‐priori identified key articles, as well as their database‐specific controlled vocabulary. These terms were supplemented with input from subject experts and keywords highlighted in Downs et al. (2020); HLPE (2017); and Turner et al. (2018). We developed a comprehensive search strategy combining subject‐heading and text word searches for the database CAB Abstracts in the Web of Science platform (see Supporting Information: Appendix 1 for full search strategy) that was searched in April 2024. This search will be adapted and run in the following databases:

**Table:** 

Database name	Platform	Date coverage
PubMed	PubMed	1946—present
Web of Science Core Collection, including SCI‐EXPANDED, SSCI, AHCI, CPCI‐S, CPCI‐SSH, BKCI‐S, BKCI‐SSH, ESCI, CCR‐EXPANDED, IC	Web of Science	1900—present
CABI: CAB Abstracts	Web of Science	1910—present
Food Science and Technology Abstracts (FSTA)	Web of Science	1969—present
Scopus	Scopus	1788—present
ProQuest Dissertations and Theses Global	ProQuest	1743—present
Database of African Theses and Dissertations—Research (DATAD‐R):	DATAD‐R	2005—present

The reference lists of included studies (including systematic reviews) will be reviewed, and all relevant studies that were not retrieved by the initial search will be screened according to the procedures described below. In addition, we will review any articles that cite our included studies (forward citations), and we will screen any relevant studies. We will use Google Scholar to identify forward citations.

Further relevant individuals and identified organizations will be contacted for information about unpublished work or ongoing studies to enable its possible inclusion at the updated review stage. Authors from relevant studies selected through the process will be contacted to obtain any additional information of the studies and to identify additional research and data that are ongoing or unpublished. To enhance precision and reduce the impact of reporting bias, study authors will be contacted for clarification of data and seek key unpublished information that may be lacking from reports of included studies.

The search process, including the search of gray literature and supplementary searches, will be conducted, documented, and reported so as to meet the standards for Campbell systematic reviews as described in MacDonald et al. (2024).

Step 3: Study selection.

### 2.3 Population

All studies that involve persons classified as pastoralists or nomadic pastoralists, male and female aged 2 years and above will be included. Pastoralists are a group of people who depend mainly on herds of domesticated animals, such as sheep, camel, goat, and cattle, for their livelihood (Barfield 2011). Pastoralists are characterized by their reliance on natural pastures, often moving from place to place in search of water and pasture for their livestock and live on marginal lands that habitually lack advanced infrastructure and logistics (McKune et al. 2015; Scanes 2018; Scialabba 2022). Nomads are people without a permanent settlement and instead move from place to place with their families and belongings in search of resources, such as food, water, and shelter. Nomads adapt to new environments and can hunt wild animals and plants or keep some animals for food. This means that nomads do not necessarily have to own livestock. Nomadic pastoralists' livelihoods depend on livestock herding and they move from region to region to find pastures for their animals. Pastoral nomadism is commonly found where climatic conditions produce seasonal pastures but cannot support sustained crop production (Ansari‐Renani et al. 2013; Barfield 2011). Studies of people not classified as pastoralists or nomadic pastoralists will be excluded. Studies involving pastoralists children under 2 years will be excluded even if they focus on the food environment, diets, and nutrition outcomes.

### 2.4 Concept

The review will consider studies on domains of the food environment and their influence on food choice, dietary intake, and nutrition outcomes of pastoralists in Africa. Particularly the relationship between the food environment or one of the food environment domains with at least one of the other aspects, namely food choice, dietary intake, and nutrition outcomes. Further, papers on description and analysis of the food environment will be included. The food environment domains, characteristics, and food choice motives will be informed by various recent food environment frameworks and multi‐country studies (Constantinides et al. 2021; Downs et al. 2022; Karanja et al. 2022; Turner et al. 2018, 2020).

### 2.5 Context

All countries in Africa will be considered in this review irrespective of their income status. If the study involves Africa and a non‐African country, the study will be included if data relevant to the African country is separate and can be expunged from the rest of the non‐African countries.

We will search sixteen academic databases and three gray literature repositories, and we will export the results of those searches as RIS files (where possible) and upload the results into the citation manager Endnote (X9.3.3). Search results will then be exported as single RIS files (one per information source) and imported into the web‐based screening platform Covidence (covidence.org). We will use Covidence's automatic de‐duplicator to remove duplicates, and any additional duplicates will be removed manually in the screening process. The MECCIR principles will be used to guide the selection process. Screening will be done in two stages by two members of the team using Covidence. In the first stage, screeners will assess titles and abstracts of the search results according to established eligibility criteria. If a study meets a single exclusion criterion, it will be excluded. Any study that is not excluded on these grounds will move to the second stage. At this stage, two screeners will independently assess the full text of each study. Any study that meets all of the inclusion criteria will move to the extraction phase. The ineligible articles will be documented, with reasons for ineligibility, which will be included in the review article. In case of any discrepancies, discussions between the two members will be held to build consensus.

Step 4: Charting the data

Data extraction will be performed using the framework developed by Arksey and O'Malley (2005) and as per the selection criteria and in line with the PCC approach. The data extraction form, Supporting Information: Appendix 2, will be pretested by two team members by sampling 10% of the studies included to certify that the procedure and capturing of information is well comprehended between them. Any clarifications and corrections/revisions on the extraction form will be done, if necessary, before the actual data extraction exercise commences. Inter‐rater reliability will be attained by randomly sampling 20% of the studies screened by the two reviewers and comparing them. Any differences will be discussed between the two reviewers for consensus, or by engaging a third reviewer. In case there is a difference in half of the 20% of the studies, another 10% will be randomly selected and reviewed until agreement is reached.

To reduce selection bias, eligibility criteria will be strictly followed by the two reviewers. Risk of bias assessment will not be performed given the nature of the scoping review and its main aim being to identify and chart evidence available on given concepts of the research (Aromataris et al. 2024).

Step 5: Collating, summarizing, and reporting results

This process will be guided by Tricco et al. (2018), using a final checklist, which comprises 20 essential reporting items and two optional items (Supporting Information: Appendix 3). This checklist will ensure that the process of study selection is logically reported and represented in the PRISMA flow diagram (Peters et al. 2017). At stage 5, three distinct steps as recommended by (Levac et al. 2010) will be followed, namely: analyzing the data, reporting results, and applying meaning to the results. The steps will be in line with the PCC framework aim and will answer the study questions.

A descriptive numerical summary and a thematic analysis will be conducted to evaluate and categorize quantitative and qualitative data. Qualitative data will be analyzed and themed according to the research questions. Themes on the food environment domains, food choice, dietary intake, and nutrition outcomes will be identified and summarized. Food environment domains will be triangulated with food choice motives, dietary intake, and nutrition outcomes, to identify potential research gaps.

## Author Contributions

Esther Omosa, Paula Dominguez‐Salas, and Inge D. Brouwer conceptualized and designed the review. Matthew Kibbee and Natasha Bishop developed and ran the search strategy. Esther Omosa, Paula Dominguez‐Salas, Inge D. Brouwer, Francoise Cattaneo, Matthew Kibbee, and Bishop contributed to the methodology, search criteria, and analysis plan. Esther Omosa and Francoise Cattaneo performed the search and screening of the studies. Esther Omosa wrote the original draft and Matthew Kibbee, Natasha Bishop, Francoise Cattaneo, Paula Dominguez‐Salas, and Inge D. Brouwer contributed to writing and editing the protocol. All authors have read this manuscript and agreed to the published version.

## Ethics Statement

Ethical approval is not mandatory as data will be from existing publications. This scoping review findings will be part of a PhD thesis for Wageningen University and Research; the findings will be published in a peer‐reviewed journal.

## Conflicts of Interest

The authors declare no conflicts of interest.

### Peer Review

The peer review history for this article is available at https://www.webofscience.com/api/gateway/wos/peer-review/10.1002/cl2.70030.

## Supporting information

Supporting information.

Supporting information.

## Data Availability

I confirm that I will make available data from this study.
