# Supplementary material for: PROTOCOL: Food Environment, Food Choice, Diets, and Nutrition Outcomes of Pastoralists in Africa: Scoping Review Protocol
Source: Campbell Syst Rev. 2025 Mar 10;21(1):e70030. doi: 10.1002/cl2.70030 (PMC11891923; doi:10.1002/cl2.70030)
Supplement: Supplementary file 1 — Supporting information. [file CL2-21-e70030-s001.docx]

| **Database:** CABI: CAB Abstracts  **Platform:** Web of Science (Clarivate Analytics)  **Note 1:** Exact Search: ON for all searches  **Note 2:** TS (Topic) field code in this platform includes many different fields beyond title and abstract. Therefore, textword searches were limited to TI and AB field codes to improve precision. | | |
| --- | --- | --- |
| **Search No.** | **Search String** | **Search Description** |
| 1. | DE=("food environment" OR "food deserts" OR "food access" OR "food security" OR "nutrition security" OR "food marketing" OR "food merchandising" OR "food packaging" OR "food prices" OR "food processing" OR "food processing quality" OR "food purchasing") | Food environment (personal and external): subject headings |
| 2. | TI=((food* NEAR/2 environment*) OR "food desert*" OR "food swamp*" OR "obesogenic environment*" OR "nutrition* environment*" OR "food system*" OR ((food* OR fruit* OR vegetable*) NEAR/5 (avail* OR access* OR qual* OR composition OR accept*)) OR "food secur*" OR "food insecur*" OR "nutrition secur*" OR "nutrition insecur*" OR "food* process*" OR ((distance OR transport* OR "time use" OR "time allocation") NEAR/5 food) OR "food litera*" OR "nutrition* qual*" OR ("nutrient dense" NEAR/2 food*) OR "food culture*" OR (food* NEAR/2 tradition*) OR "cultural food*" OR "prestige food*" OR "food prestige" OR "food taboo*" OR "food context*" OR "vendor* propert*" OR "product* propert*" OR "shelf-life" OR "food* packag*" OR "food regulat*" OR "food* promot*" OR "promot* info*" OR "nutr* info*" OR "nutr* promot*" OR brand* OR advertis* OR sponsor* OR "food* label*" OR "food* polic*" OR "open* hour*" OR "vendor* servic*" OR "vendor* typolog*" OR ((distance OR proximity) NEAR/3 market*) OR (vendor* NEAR/2 access*) OR "food afford*" OR ("purchas* power" NEAR/5 food*) OR "buying power" OR "food allow*" OR "sustainable supply chain*" OR "sustainable food package*" OR "local supply chain*" OR "price elasticit*" OR "social capital" OR "social network*" OR "food aid" OR "food assistance" OR (food NEAR/2 transfer*) OR (food NEAR/2 barter*) OR (food NEAR/2 (share$ OR sharing)) OR "community food" OR "food remit*") OR (AB=((food* NEAR/2 environment*) OR "food desert*" OR "food swamp*" OR "obesogenic environment*" OR "nutrition* environment*" OR "food system*" OR ((food* OR fruit* OR vegetable*) NEAR/5 (avail* OR access* OR qual* OR composition OR accept*)) OR "food secur*" OR "food insecur*" OR "nutrition secur*" OR "nutrition insecur*" OR "food* process*" OR ((distance OR transport* OR "time use" OR "time allocation") NEAR/5 food) OR "food litera*" OR "nutrition* qual*" OR ("nutrient dense" NEAR/2 food*) OR "food culture*" OR (food* NEAR/2 tradition*) OR "cultural food*" OR "prestige food*" OR "food prestige" OR "food taboo*" OR "food context*" OR "vendor* propert*" OR "product* propert*" OR "shelf-life" OR "food* packag*" OR "food regul*" OR "food* promot*" OR "promot* info*" OR "nutr* info*" OR "nutr* promot*" OR brand* OR advertis* OR sponsor* OR "food* label*" OR "food* polic*" OR "open* hour*" OR "vendor* servic*" OR "vendor* typolog*" OR ((distance OR proximity) NEAR/3 market*) OR (vendor* NEAR/2 access*) OR "food afford*" OR ("purchas* power" NEAR/5 food*) OR "buying power" OR "food allow*" OR "sustainable supply chain*" OR "sustainable food package*" OR "local supply chain*" OR “price elasticit*” OR “"price elasticit*" OR "social capital" OR "social network*" OR "food aid" OR "food assistance" OR (food NEAR/2 transfer*) OR (food NEAR/2 barter*) OR (food NEAR/2 (share$ OR sharing)) OR "community food" OR "food remit*")) | Food environment (personal and external): textwords |
| 3. | DE=("oceans" OR "wild foods" OR "game meat" OR "fishing") | Food environment (wild): subject headings |
| 4. | TI=("wild food*" OR (wild NEAR/2 edible) OR ocean$ OR coast* OR fisher* OR fishing OR forest* OR forag* OR hunting OR hunter* OR "game meat*" OR "bush meat" OR (food* NEAR/2 (traditional* OR indigenous OR underutilized OR "under utilized"))) OR (AB=("wild food*" OR (wild NEAR/2 edible) OR ocean$ OR coast* OR fisher* OR fishing OR forest* OR forag* OR hunting OR hunter* OR "game meat*" OR "bush meat" OR (food* NEAR/2 (traditional* OR indigenous OR underutilized OR "under utilized")))) | Food environment (wild): textwords |
| 5. | DE=("agriculture" OR "animal husbandry" OR "crop production" OR "farming" OR "farms" OR "food production" OR "gardens" OR "home gardens" OR "aquaculture" OR "fishing") | Food environment (cultivated): subject headings |
| 6. | TI=("own production" OR (("crop production" OR farm* OR agricultur* OR livestock OR animal*) NEAR/3 (divers* OR variet*)) OR "crop spec* rich*" OR "livestock owners*" OR "animal-source* food*" OR "food produc*" OR (home* NEAR/2 garden*) OR (homestead NEAR/2 (produc* OR food* OR farm*)) OR (prepar* NEAR/2 food*) OR cook* OR "crop qual*" OR "seasonal food*" OR "sustainable agriculture" OR "environmental* sustain*" OR biodivers* OR agrobiodivers* OR "environment* footprint" OR "carbon footprint*" OR "climate resilien*" OR aquaculture* OR fishing OR fisher* OR orchard* OR (jungle* NEAR/4 food*) OR "neglected species" OR "underutilized species") OR (AB=("own production" OR (("crop production" OR farm* OR agricultur* OR livestock OR animal*) NEAR/3 (divers* OR variet*)) OR "crop spec* rich*" OR "livestock owners*" OR "animal-source* food*" OR "food produc*" OR (home* NEAR/2 garden*) OR (homestead NEAR/2 (produc* OR food* OR farm*)) OR (prepar* NEAR/2 food*) OR cook* OR "crop qual*" OR "seasonal food*" OR "sustainable agriculture" OR "environmental* sustain*" OR biodivers* OR agrobiodivers* OR "environment* footprint" OR "carbon footprint*" OR "climate resilien*" OR aquaculture* OR fishing OR fisher* OR orchard* OR (jungle* NEAR/4 food*) OR "neglected species" OR "underutilized species")) | Food Environment (Cultivated) |
| 7. | DE=("street markets" OR "informal sector") | Food Environment (Informal Market): subject headings |
| 8. | TI=(((food* OR beverage*) AND ((street OR mobile OR informal OR grey OR gray OR unregulated OR unlicen?ed OR "un regulated" OR "un licen?ed") NEAR/2 (vend* OR market* OR seller* OR trade* OR stall OR stalls OR cart OR carts OR hawker*))) OR (informal NEAR/1 (trade* OR market* OR vend* OR food* OR beverage*)) OR "street food*" OR "street vend*" OR "street market*" OR "street trad*" OR "public eating" OR "wet market*" OR (((food OR beverage*) NEAR/2 market*) AND (street OR informal OR unlicen?ed OR unregulated)) OR ((food OR beverage*) NEAR/2 (vendor* OR street OR informal OR unlicen?ed OR unregulated)) OR (((food* OR beverage*) NEAR/2 seller*) AND (street OR informal OR unlicen?ed OR unregulated)) OR (((food OR beverage*) NEAR/2 retail*) AND (street OR informal OR unlicen?ed OR unregulated)) OR (((food OR beverage*) NEAR/2 stall*) AND (street OR informal OR unlicen?ed OR unregulated))) OR (AB=(((food* OR beverage*) AND ((street OR mobile OR informal OR grey OR gray OR unregulated OR unlicen?ed OR "un regulated" OR "un licen?ed") NEAR/2 (vend* OR market* OR seller* OR trade* OR stall OR stalls OR cart OR carts OR hawker*))) OR (informal NEAR/1 (trade* OR market* OR vend* OR food* OR beverage*)) OR "street food*" OR "street vend*" OR "street market*" OR "street trad*" OR "public eating" OR "wet market*" OR (((food OR beverage*) NEAR/2 market*) AND (street OR informal OR unlicen?ed OR unregulated)) OR ((food OR beverage*) NEAR/2 (vendor* OR street OR informal OR unlicen?ed OR unregulated)) OR (((food* OR beverage*) NEAR/2 seller*) AND (street OR informal OR unlicen?ed OR unregulated)) OR (((food OR beverage*) NEAR/2 retail*) AND (street OR informal OR unlicen?ed OR unregulated)) OR (((food OR beverage*) NEAR/2 stall*) AND (street OR informal OR unlicen?ed OR unregulated)))) | Food Environment (Informal Market) |
| 9. | DE=("supermarkets" OR "markets" OR "food stores" OR "school meals" OR "school breakfasts" OR "school lunches" OR "food stores" OR "grocers") | Food Environment (Formal Market): subject headings |
| 10. | TI=("food vendor*" OR "food outlet*" OR ((retail OR market$) NEAR/4 (food* OR dairy)) OR ((value OR price* OR cost OR expenditure* OR budget*) NEAR/2 food*) OR supermarket* OR ((food* OR beverage* OR produce OR fruit OR vegetable OR meat) NEAR/3 (wholesaler* OR "whole saler*" OR processor* OR retailer* OR seller* OR "consumer cooperative*" OR "consumer co operative*")) OR ((school OR hospital* OR prison OR institution*) NEAR/2 (meal* OR snack* OR lunch* OR food* OR breakfast*)) OR restaurant* OR ((food* OR beverage* OR juice* OR water OR soda* OR meal* OR eat* OR consum*) NEAR/3 ((out* OR away) NEAR/2 home*)) OR "take away" OR takeaway OR "carry out" OR carryout OR grocer* OR ((food* OR beverage* OR produce OR fruit OR vegetable OR meat OR dairy OR corner OR convenience) NEAR/3 (shop OR shops OR store*)) OR ((microenterprise* OR "micro enterprise*" OR trade* OR firm* OR microfirm* OR "micro firm*") NEAR/3 (food OR beverage*)) OR "superstore*" OR "super store*" OR "coop" OR "co op") OR ( AB=("food vendor*" OR "food outlet*" OR ((retail OR market$) NEAR/4 (food* OR dairy)) OR ((value OR price* OR cost OR expenditure* OR budget*) NEAR/2 food*) OR supermarket* OR ((food* OR beverage* OR produce OR fruit OR vegetable OR meat) NEAR/3 (wholesaler* OR "whole saler*" OR processor* OR retailer* OR seller* OR "consumer cooperative*" OR "consumer co operative*")) OR ((school OR hospital* OR prison OR institution*) NEAR/2 (meal* OR snack* OR lunch* OR food* OR breakfast*)) OR restaurant* OR ((food* OR beverage* OR juice* OR water OR soda* OR meal* OR eat* OR consum*) NEAR/3 ((out* OR away) NEAR/2 home*)) OR "take away" OR takeaway OR "carry out" OR carryout OR grocer* OR ((food* OR beverage* OR produce OR fruit OR vegetable OR meat OR dairy OR corner OR convenience) NEAR/3 (shop OR shops OR store*)) OR ((microenterprise* OR "micro enterprise*" OR trade* OR firm* OR microfirm* OR "micro firm*") NEAR/3 (food OR beverage*)) OR "superstore*" OR "super store*" OR "coop" OR "co op")) | Food Environment (Formal Market) |
| 11. | #1 OR #2 OR #3 OR #4 OR #5 OR #6 OR #7 OR #8 OR #9 OR #10 | Food Environment |
| 12. | DE=("caloric intake" OR "energy intake" OR "food intake" OR "nutrient intake" OR "vitamins" OR "nutrients" OR "trace elements" OR "minerals") | Dietary intake outcomes: subject headings |
| 13. | TI=(((Food* OR nutrient* OR energy OR dietary OR fruit* OR vegetable* OR grain* OR root* OR tuber* OR pulse* OR legume* OR nut* OR seed* OR dairy* OR meat* OR poultry OR fish* OR shellfish* OR seafood OR egg* OR "leafy green*" OR beef* OR pork OR chicken* OR goat* OR cheese* OR yog?urt OR milk* OR cereal* OR bean*) NEAR/2 (intake* OR diet$ OR consum*)) OR micronutrient* OR "micro nutrient*" OR "consumption pattern*" OR kcal* OR "vitamin A*" OR "Vitamin C*" OR "vitamin D*" OR "vitamin E" OR "vitamin K*" OR thiamine* OR riboflavin* OR niacin*OR "pantothenic acid*" OR biotin* OR "vitamin B6*"OR B6 OR "b 12" OR b12 OR folate* OR "folic acid" OR iron OR calcium OR zinc) OR (AB= (((Food* OR nutrient* OR energy OR dietary OR fruit* OR vegetable* OR grain* OR root* OR tuber* OR pulse* OR legume* OR nut* OR seed* OR dairy* OR meat* OR poultry OR fish* OR shellfish* OR seafood OR egg* OR "leafy green*" OR pork OR beef* OR chicken* OR goat OR cheese* OR yog*urt OR milk* OR cereal* OR bean*) NEAR/2 (intake* OR diet$ OR intake* OR consum*)) OR micronutrient* OR "micro nutrient*" OR "consumption pattern*" OR kcal* OR "vitamin A*" OR "Vitamin C*" OR "vitamin D*" OR "vitamain E" OR "vitamin K*" OR thiamine* OR riboflavin* OR niacin*OR "pantothenic acid*" OR biotin* OR "vitamin B6*" OR B6 OR "vitamin B 12*" OR b12 OR folate* OR "folic acid" OR iron OR calcium OR zinc)) | Dietary intake outcomes: textwords |
| 14. | DE=("obesity" OR "body fat" OR "body mass index" OR "overweight" OR "thinness" OR "underweight" OR "undernutrition" OR "malnutrition" OR "deficiency diseases" OR "hunger" OR "protein energy malnutrition" OR "nutritional anaemia" OR "anaemia" OR "nutrient deficiencies" OR "nutritional state") | Nutritional outcomes: subject headings |
| 15. | TI=(undernourish* OR "under nourish*" OR undernutrition OR "under nutrition" OR obes* OR "over weight" OR overweight OR malnour* OR malnutrition OR ((vitamin* OR mineral* OR micronutrient* OR "micro nutrient*" OR nutrient*) NEAR/2 deficienc*) OR diarrh$ea OR "metabolic syndrome*" OR BMI OR "body mass index" OR MUAC OR "Mid upper arm circumference" OR anaemi* OR anemi* OR "nutri* status*" OR ((waist OR hip* OR head) NEAR/2 circumference*) OR stunting OR wasting OR thin OR thinness OR underweight OR "under weight" OR "height for age" OR "weight for height" OR "waist hip ratio" OR "waist to hip ratio" OR diabet*) OR (AB=(undernourish* OR "under nourish*" OR undernutrition OR "under nutrition" OR obes* OR "over weight" OR overweight OR malnour* OR malnutrition OR ((vitamin* OR mineral* OR micronutrient* OR "micro nutrient*" OR nutrient*) NEAR/2 deficienc*) OR diarrh$ea OR "metabolic syndrome*" OR BMI OR "body mass index" OR MUAC OR "Mid upper arm circumference" OR anaemi* OR anemi* OR "nutri* status*" OR ((waist OR hip* OR head) NEAR/2 circumference*) OR stunting OR wasting OR thin OR thinness OR underweight OR "under weight" OR "height for age" OR "weight for height" OR "waist hip ratio" OR "waist to hip ratio" OR diabet*)) | Nutritional outcomes: textwords |
| 16. | DE=("food preferences" OR "feeding preferences") | Food choice outcomes: subject headings |
| 17. | TI=("food choice*" OR (food* NEAR/3 ("decision making" OR desir* OR prefer* OR attitude*)) OR Health* OR convenience* OR price* OR "sensory appeal*" OR "natural content*" OR mood* OR familiarity* OR ethic* OR concern* OR weight* OR control* OR tast* OR "food knowledge" OR "food skill*") OR (AB=("food choice*" OR (food* NEAR/3 ("decision making" OR desir* OR prefer* OR attitude*)) OR Health* OR convenience* OR price* OR "sensory appeal*" OR "natural content*" OR mood* OR familiarity* OR ethic* OR concern* OR weight* OR control* OR tast* OR "food knowledge" OR "food skill*")) | Food choice outcomes: textwords |
| 18. | #12 OR #13 OR #14 OR #15 OR #16 OR #17 | Outcomes |
| 19. | DE=("pastoralism" OR "pastoral society" OR "agropastoral systems" OR "silvopastoral systems" OR "nomadism" OR "transhumance") | Pastoralist: subject headings |
| 20. | (TI=(pastoralist* OR agropastoralist* OR "agro pastoralist*" OR silvopastoralist* OR "silvo pastoralist*" OR seminomad* OR "semi nomad*" OR nomad* OR transhumance OR "distant-pastures husband*" OR ((agropastoral* OR silvopastoral* OR pastoral*) NEAR/2 (people OR person OR communit* OR child* OR adult* OR farmer* OR herder* OR men OR wom?n OR population* OR household* OR family OR families)))) OR AB=(pastoralist* OR agropastoralist* OR "agro pastoralist*" OR silvopastoralist* OR "silvo pastoralist*" OR seminomad* OR "semi nomad*" OR nomad* OR transhumance OR "distant-pastures husband*" OR ((agropastoral* OR silvopastoral* OR pastoral*) NEAR/2 (people OR person OR communit* OR child* OR adult* OR farmer* OR herder* OR men OR wom?n OR population* OR household* OR family OR families))) | Pastoralist: textwords |
| 21. | #19 OR #20 | Pastoralist |
| 22. | DE=("ACP countries" OR "Francophone Africa" OR "Africa" OR "West Africa" OR "Africa South of Sahara" OR "Anglophone Africa" OR "East Africa" OR "Central Africa" OR "North Africa") | Africa: subject headings |
| 23. | TI=(africa* OR algeria* OR angola* OR benin OR botswana* OR "burkina faso" OR burundi* OR "cabo verde" OR "cape verde" OR cameroon* OR chad OR comoros OR congo* OR "cote d'ivoire" OR "ivory coast" OR ivorian OR djibouti OR egypt* OR "equatorial guinea*" OR eritrea OR eswatini OR ethiopia* OR gabon* OR gambia* OR ghana* OR guinea OR guinean OR "guinea-bissau" OR kenya* OR lesotho OR liberia* OR libya* OR madagascar* OR malawi* OR mali OR malian OR mauritania* OR mauritius OR morocc* OR mozambique OR namibia* OR niger OR nigeria* OR rwanda* OR "sao tome" OR principe OR senegal* OR seychelles OR "sierra leone" OR somalia* OR "south africa*" OR sudan OR sudanese OR tanzania* OR togo OR togolese OR tunisia* OR uganda* OR zambia* OR zimbabwe*) OR (AB=(africa* OR algeria* OR angola* OR benin OR botswana* OR "burkina faso" OR burundi* OR "cabo verde" OR "cape verde" OR cameroon* OR chad OR comoros OR congo* OR "cote d'ivoire" OR "ivory coast" OR ivorian OR djibouti OR egypt* OR "equatorial guinea*" OR eritrea OR eswatini OR ethiopia* OR gabon* OR gambia* OR ghana* OR guinea OR guinean OR "guinea-bissau" OR kenya* OR lesotho OR liberia* OR libya* OR madagascar* OR malawi* OR mali OR malian OR mauritania* OR mauritius OR morocc* OR mozambique OR namibia* OR niger OR nigeria* OR rwanda* OR "sao tome" OR principe OR senegal* OR seychelles OR "sierra leone" OR somalia* OR "south africa*" OR sudan OR sudanese OR tanzania* OR togo OR togolese OR tunisia* OR uganda* OR zambia* OR zimbabwe*)) | Africa: textwords |
| 24. | #22 OR #23 |  |
| **25.** | **#11 AND #18 AND #21 AND #24** |  |
|  | **Date filter applied: 2000-01-01 - present** | **Date filter** |

**References**

The food environment searches (#1-10) were based on the frameworks in the following papers.

Downs, S. M., Ahmed, S., Fanzo, J., & Herforth, A. (2020). Food Environment Typology: Advancing an Expanded Definition, Framework, and Methodological Approach for Improved Characterization of Wild, Cultivated, and Built Food Environments toward Sustainable Diets. *Foods*, *9*(4), 532. <https://www.mdpi.com/2304-8158/9/4/532>

Turner, C., Kalamatianou, S., Drewnowski, A., Kulkarni, B., Kinra, S., & Kadiyala, S. (2019). Food Environment Research in Low- and Middle-Income Countries: A Systematic Scoping Review. *Advances in nutrition*, *11*(2), 387-397. <https://doi.org/10.1093/advances/nmz031>

The informal market food environment searches (#7 and #8) are adapted from the search in the following paper.

Kibbee, M. R., Ambikapathi, R., & Sirwatka, A. (2023). *Informal Employment in the Food Environment in Low- and Middle-Income Countries: A Scoping Review*. <https://doi.org/10.17605/OSF.IO/YMK3T>

The dietary intake searches (#12 and #13) derived search terms in part from the food group frameworks in the following papers.

Norde, M.M., Bromage, S., Marchioni, D.M.L. *et al.* The global diet quality score as an indicator of adequate nutrient intake and dietary quality – a nation-wide representative study. *Nutr J* **23**, 42 (2024). <https://doi.org/10.1186/s12937-024-00949-x>

Torheim LE, Ouattara F, Diarra MM, Thiam FD, Barikmo I, Hatløy A, Oshaug A. Nutrient adequacy and dietary diversity in rural Mali: association and determinants. Eur J Clin Nutr. 2004 Apr;58(4):594-604. doi: 10.1038/sj.ejcn.1601853. PMID: 15042127.
